# Exploring the Genetic Diversity of *Mycoplasma hyopneumoniae* in Pigs with Pneumonia and Pleurisy at Slaughter

**DOI:** 10.3390/microorganisms12101988

**Published:** 2024-09-30

**Authors:** Ana Karolina Panneitz, Eduarda Ribeiro Braga, Fernando Antonio Moreira Petri, Jean Carlo Olivo Menegatt, David Driemeier, Dominiek Maes, Luís Guilherme de Oliveira

**Affiliations:** 1 Swine Medicine Laboratory, School of Agricultural and Veterinary Sciences, São Paulo State University (Unesp), Jaboticabal 14884-900, Brazil; ana.panneitz@unesp.br (A.K.P.); eduarda.braga@unesp.br (E.R.B.); fernando.petri@unesp.br (F.A.M.P.); 2 Veterinary Pathology Department, Federal University of Rio Grande do Sul (UFRGS), Porto Alegre 91540-000, Brazil; menegattjean2@gmail.com (J.C.O.M.); ddriemeier@gmail.com (D.D.); 3 Unit of Porcine Health Management, Faculty of Veterinary Medicine, Ghent University, 9820 Merelbeke, Belgium; dominiek.maes@ugent.be

**Keywords:** alleles, BALT hyperplasia, MLST, pneumonia, porcine enzootic pneumonia, respiratory disease

## Abstract

*Mycoplasma* (*M.*) *hyopneumoniae* is the key pathogen of the porcine respiratory disease complex (PRDC) and contributes to pleurisy in pigs. Due to its limited metabolism and laborious cultivation, molecular tools are useful for diagnosis. This study investigated the genetic diversity of *M. hyopneumoniae* in slaughter pigs with pneumonia and pleurisy, and it assessed co-infections by *Pasteurella multocida* type A (PM), *Actinobacillus pleuropneumoniae* (APP), and swine influenza virus A (sIVA). Lungs (*n* = 70) with different pleurisy scores and lesions compatible with *M. hyopneumoniae* infection were collected for convenience. Macroscopic and microscopic evaluations were performed. *M. hyopneumoniae* was detected using qPCR, and MLST was used for genetic characterization. Co-infections with PM and APP were also evaluated by qPCR, while the immunohistochemistry assessed sIVA infection. All lungs were positive for *M. hyopneumoniae*. Histopathology confirmed *M. hyopneumoniae*-associated lesions. MLST characterization was possible in 25 lungs and revealed 10 distinct allelic profiles, with none matching known sequence types in the public database. Co-infections were detected in 40% of the samples with APP and 32% with PM, with 12% showing both pathogens and 52% of the samples presenting microscopic lesions compatible with sIVA infection. The diverse genetic profiles found underscore the need for research on isolation and potential pathogenic variations.

## 1. Introduction

Bacteria belonging to the genus *Mycoplasma* spp. are the smallest self-replicating microorganisms and are phenotypically distinguished from other bacteria by the absence of a cell wall [1]. These characteristics contribute to the difficulty of in vitro cultivation of the bacteria belonging to this genus. *Mycoplasma* (*M.*) *hyopneumoniae* is the etiologic agent of porcine enzootic pneumonia (PEP) and one of the primary agents of the porcine respiratory disease complex (PRDC) [2]. The clinical manifestation of infection is typically characterized as chronic bronchopneumonia, with a non-productive cough being the most characteristic sign, and it is most evident in the growth and finishing phases [3]. The losses linked to *M. hyopneumoniae* infection are associated with a reduction in zootechnical indices, increased susceptibility to secondary infections, which can lead to higher antibiotic use and carcasses being condemned in the slaughterhouse [2,4]. Ferraz et al. [5] estimated an economic impact of USD 6.55 per affected pig slaughtered due to *M. hyopneumoniae* infection. Definitive diagnosis of the disease is achieved by necropsy, histopathology, and molecular assays [6]. *M. hyopneumoniae* is also often detected in cases of pleurisy in slaughter pigs, although the pathogen itself does not induce this lesion. The microenvironment resulting from the lung lesions likely provides favorable conditions for co-infections like *Pasteurella multocida type* A (PM) and *Actinobacillus pleuropneumoniae* (APP) [7]. Pleurisy is an important parameter for evaluation in slaughterhouses because it can lead to the condemnation of carcasses, generating a higher economic impact on the industry and challenging diagnoses [8,9]. Furthermore, previous studies indicate that the prevalence of this lesion type in Brazilian herds is higher than 10% [4,8].

Knowledge about the pathogenicity and virulence factors of *M. hyopneumoniae* is scarce because of the in vitro growth characteristics [1]. However, there are differences in the clinical course of the disease and the severity of the lesions, which might be related to these characteristics [10]. Because it is a difficult bacterium to cultivate, different molecular biology tools are used for the epidemiological characterization of *M. hyopneumoniae*. Multi-locus sequence typing (MLST) and multi-locus variable number tandem repeats (MLVA) are the most widely used techniques due to their high power in discriminating variants. There are also other methods for genotyping, such as pulsed-field gel electrophoresis (PFGE), random amplified polymorphic DNA (RAPD), restriction fragment length polymorphism (RFLP), and high-throughput sequencing [10,11]. Technological advances in next-generation sequencing platforms have the capacity to improve the accuracy of genetic diversity studies of microorganisms, but more studies are still needed to create robust databases [12].

The MLST technique is well established and standardized for characterizing different species of bacteria. For *M. hyopneumoniae*, several loci have been evaluated for this technique in both putative genes and house-keeping genes. Currently, for this pathogen, it analyzes three target genes (*adk*, *rpoB*, and *tipA*) [13]. One advantage of MLST is its public online database for sharing and comparing results. Thus far, the MLST technique can discriminate 392 isolates in 469 sequence types (STs) (https://pubmlst.org/organisms/mycoplasma-hyopneumoniae (accessed on 2 August 2024)).

Some studies on the genetic diversity of *M. hyopneumoniae* in Brazil have shown high genetic diversity [14,15,16]. However, these studies used another tool for genetic characterization, which is a barrier to comparing results. Takeuti et al. (2017) [14] and dos Santos et al. (2015) [15] used the MLVA technique to characterize *M. hyopneumoniae*, while Assao et al. (2019) [16] assessed the similarity according to the presence of some of the genes studied. Balestrin et al. [17] used the MLST approach to investigate the genetic diversity of *M. hyopneumoniae* strains in Brazil, but this study found low genetic diversity between strains and also no relation with the geographical location. In that study, five STs were identified as circulating in Brazil, and most of the samples were identified as belonging to the ST-69 group (23-33-26). The contrast in results emphasizes the necessity of increasing the number of studies on this topic to provide data for comparisons.

Based on the high prevalence, the economic impact, and the differences in virulence and severity of the disease associated with the pathogen, our study investigated the genetic diversity of *M. hyopneumoniae* using the MLST technique in slaughter pigs with pneumonia and pleurisy and the occurrence of co-infections involving APP, PM, and sIVA.

## 2. Materials and Methods

### 2.1. Macroscopic Evaluation of Lungs and Sample Collection

**Figure 1:**
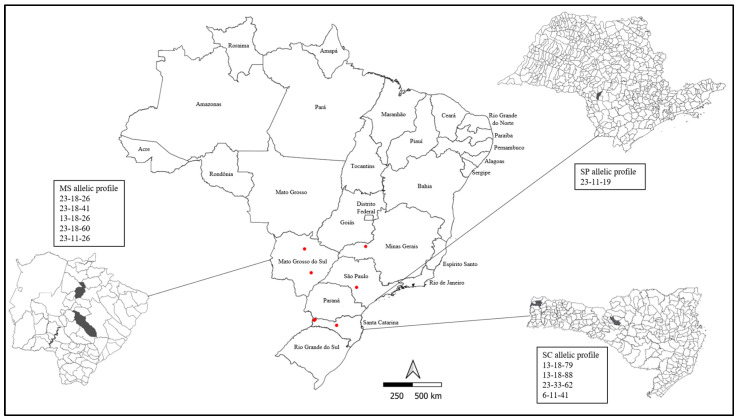
Geographical distribution of batches sampled on a Brazilian map. Red dots represent the farms’ cities. The allelic profile of *M. hyopneunoniae* from three states was evaluated. SC = Santa Catarina; MS = Mato Grosso do Sul; SP = São Paulo. The area marked in black in each state indicates the city from which the herds originated. Figure created using QGIS software version 3.4.5.

Between 2022 and 2023, the lungs of pigs were collected for convenience in a slaughterhouse located in Guariba, São Paulo, Brazil. The lungs presenting different pleurisy scores and lesions suggestive of *M. hyopneumoniae* infection (cranioventral region of the lung showing areas of consolidation with purple-to-gray discoloration). Batches were sampled from four important pig-producing states in Brazil (Figure 1), including seven distinct cities: Uberlândia (Minas Gerais), São Gabriel do Oeste, Campo Grande (Mato Grosso do Sul), Cerqueira César (São Paulo), Guarujá do Sul, São José dos Cedros, and Videira (Santa Catarina).

After evisceration, the lungs were classified according to the severity of pleurisy using the Slaughterhouse Pleurisy Evaluation System (SPES) proposed by Dottori et al. (2007) [18]. Briefly, the classification is based on the severity of the pleurisy lesions, with a score of 0 in the absence of pleurisy, a score 1 for pleurisy affecting the cranioventral portion of the lung, a score of 2 indicating discrete unilateral pleurisy in the caudal lobes, a score 3 indicating discrete bilateral pleurisy in the caudal lobes or extensive unilateral pleurisy in the caudal lobe, and a score of 4 indicating an extensive bilateral lesion with bilateral pleurisy in the caudal lobes. From each batch, 10 pigs were sampled, with 2 pigs per pleurisy score (*n* = 70).

The severity of the pneumonia lesions was assessed by following the methodology proposed by Madec and Kobisch (1982) [19]. Briefly, each lung lobe received a score from 0 to 4: (0) no lesion; (1) lesion affecting <25% of the lobe surface; (2) lesion affecting 25–49% of the surface; (3) lesion affecting 50–74% of the surface; and (4) lesion affecting ≥75% of the surface. The points per lobe were summed to provide an overall area lung score (0–28).

After the gross evaluation, fragments of lung tissue were collected from the apical-cardiac-diaphragmatic lobes at the edge between the affected and non-affected tissue (including bronchi) for microscopic and molecular analysis. The samples for PCR analyses were stored in duplicate for molecular assays in RNAse- and DNAse-free cryogenic tubes and stored in a freezer at −80 °C until laboratory analysis. The lung samples for histopathological and immunohistochemical analysis were stored in 10% buffered formalin at a 1:10 tissue/formalin ratio. All procedures described in this study were approved by the Animal Use Ethics Committee (CEUA) of FCAV/Unesp-Campus Jaboticabal under protocol number 001113/23.

### 2.2. DNA Extraction and Conventional PCR (cPCR) for Endogenous Mammalian Glyceraldehyde 3-Phosphate Dehydrogenase (Gapdh) Gene

DNA was extracted from the lung samples using an in-house Tris-HCl protocol adapted from Kuramae-Izioka (1977) [20]. The concentration and quality of the extracted DNA were measured using spectrophotometry (NanoDrop^®^ One Spectrophotometer, Thermo Fisher Scientific, Waltham, MA, USA).

To check for possible inhibitors in the extracted DNA samples and the occurrence of false negatives, all samples were tested by conventional PCR (cPCR) as described by Birkenheuer et al. (2003) [21]. This cPCR detects the presence of a fragment of the endogenous mammalian glyceraldehyde 3-phosphate dehydrogenase gene (*gapdh*). The samples were only submitted to qPCR if this gene was amplified, avoiding false-negative results due to PCR inhibitors.

### 2.3. Real-Time PCR (qPCR) for p102 Gene of M. hyopneumoniae

The extracted DNA, which was positive for the presence of the *gapdh* gene, was submitted to qPCR to detect fragments of the *p102* gene (adhesion protein) of *M. hyopneumoniae* as described by Fourour et al. (2018) [22] and adapted by Almeida et al. (2020) [23]. Table S1 describes the target genes, primers, probes, and amplicon size.

### 2.4. Molecular Characterization of M. hyopneumoniae

The multi-locus sequence typing (MLST) technique was performed as described by Mayor et al. (2008) [13] and preconized by the MLST public database (https://pubmlst.org/organisms/mycoplasma-hyopneumoniae (accessed on 16 July 2024). We amplified via cPCR three housekeeping genes: *adk* (encoding adenylate kinase), *rpoB* (coding for the b-subunit of the RNA polymerase), and *tpiA* (encodes the triosephosphate isomerase). Table S2 describes the target genes, the primers, and the amplicon size.

The cPCR-amplified products were submitted to horizontal electrophoresis in a 1.5% agarose gel stained with SYBR SAFE (10,000×) (Thermo Fisher Scientific^®^, Waltham, MA, USA) using TBE (pH 8.0) running buffer at 110 V/500 mA for 60 min. A molecular weight marker of 100 base pairs (Cellco Biotec, São Carlos, SP, Brazil) was used in each electrophoresis, and the amplified fragments were visualized in an ultraviolet light transilluminator. The amplicons were purified enzymatically using ExoSAP-IT (Thermo Fisher Scientific^®^, Waltham, MA, USA) according to the manufacturer’s instructions. The purified genetic material was quantified using spectrophotometry (NanoDrop^®^ One Spectrophotometer, Thermo Fisher Scientific^®^, Waltham, MA, USA).

Samples positive for the 3 target genes were submitted to Sanger sequencing (*n* = 25). Approximately 10–20 ng of the purified PCR product was submitted to sequencing using the same cPCR primers. The amplified products were purified with a commercial kit and sequenced using a BigDye™ Terminator v3.1 Cycle Sequencing Kit (Thermo Fisher Scientific™, Waltham, MA, USA) and ABI PRISM 310DNA Analyzer (Applied Biosystems™, Waltham, MA, USA).

Sequences were assembled, edited, and trimmed using BioNumerics version 7.6.3 (Applied Maths, Sint-Martens-Latem, Belgium). Sequence types (STs) were assigned by BioNumerics using allelic profiles in the gene order of *adk*-*rpoB*-*tpiA*. The same order was used to concatenate sequences for phylogenetic analysis, and cluster analysis was performed using the neighbor joining method. The evolutionary distances were computed using the *p*-distance method and expressed as the number of base differences per site with Molecular Evolutionary Genetics Analysis (MEGA) software (version 11; https://www.megasoftware.net/, accessed on 8 August 2024).

### 2.5. Detection of Bacterial Co-Infections by Multiplex qPCR

A subset of positive samples for 3 target genes for MLST (*n* = 25) was selected for co-infection detection. The protocols for the detection of APP and PM were previously described by Sunaga et al. (2020) [7] and Goecke et al. (2021) [24] and adapted by Petri et al. (2023) [4]. The multiplex qPCR targets were the *omlA* gene (virulence protein) and the *kmt1* gene (membrane lipoprotein) from APP and PM, respectively. Table S1 describes the target genes, primers, probes, and amplicon size.

### 2.6. Histopathological and Immunohistochemical Evaluation

Lung samples positive for MLST target genes (*n* = 25) were routinely processed and stained with hematoxylin and eosin (HE) for histopathological examination. *M. hyopneumoniae* lesions were systematically analyzed using a methodology adapted from Hansen et al. (2010) [25]. Bronchus-associated lymphoid tissue (BALT) was carefully examined and graded for the presence of hyperplasia as follows: (0) absent, in which no inflammatory infiltrate was observed in the peribronchial or peribronchiolar tissues with no evidence of lymphoid nodules; (+) mild diffuse inflammatory infiltrate of lymphocytes in the peribronchial and peribronchiolar tissues, including the alveolar septa associated with the presence of a few lymphoid nodules; (++) moderate diffuse inflammatory infiltration of lymphocytes in the peribronchial and peribronchiolar tissues, including the alveolar septa associated with a marked number of lymphoid nodules; and (+++) an extensive number of lymphoid nodules with apparent occlusion of the airway lumen.

Secondary bacterial lung lesions were classified according to airway exudates as suppurative bronchopneumonia (a marked increase of neutrophils in the alveoli, bronchi, and bronchioles); necro-suppurative bronchopneumonia (a marked increase of neutrophils in the airway associated with areas of lung necrosis); and pleuropneumonia (acute or subacute pleuropneumonia (visceral pleura thickened by intense fibrin deposition, necrotic cellular debris, and many degenerate neutrophils, as well as areas of necro-suppurative bronchopneumonia) or chronic pleuropneumonia (characterized by extensive fibrous connective tissue proliferation on the pleura, fibrin deposition, and necrotic lung areas)).

Chronic pleural lesions (chronic pleuritis) were evaluated separately, characterized by thickening of the pleura due to the proliferation of fibrous connective tissue and blood vessels, as well as mild inflammatory infiltration of lymphocytes and macrophages. Additionally, non-specific lesions such as type II pneumocyte hyperplasia and alveolar edema were evaluated for their presence or absence.

Immunohistochemistry (IHC) was applied to paraffin-embedded tissues in cases with histological lesions compatible with swine influenza virus (sIVA) infection as described by Watanabe et al. (2012) [26]. The sIVA lesions were identified based on characteristic histological findings of lymphocytic bronchointerstitial pneumonia, accompanied by necrotic, proliferative, or obliterative bronchiolitis.

### 2.7. Statistical Analysis

The variables were assessed for normality and homoscedasticity using the Shapiro–Wilk test. Descriptive statistics are presented as the mean and standard deviation for normally distributed data and as a median with a range between the first and third quartiles (IQR) when the data were not normally distributed. Parametric data were analyzed using analysis of variance (ANOVA) followed Tukey’s test for multiple comparisons of means (*p* < 0.05). Non-parametric data were analyzed using the Mann–Whitney test for pairwise comparisons of medians (*p* < 0.05). All data analyses were performed using the GraphPad Prism 10.2.2 software (La Jolla, CA, USA).

## 3. Results

### 3.1. Gross Evaluation and qPCR for the Detection of M. hyopneumoniae

The lung consolidation score ranged from 0 to 28 points. Table S3 shows the consolidation points per lung lobe, as well as the total score per lung and the score of pleurisy. The median lung consolidation score across all lungs was six points, with an interquartile range (IQR) of six (3–9). There was a statistical difference (*p* value: 0.0001) when comparing the median scores of lungs with pleurisy (7; IQR: 6 (4–10)) and without pleurisy lesions (3; IQR: 3.25 (1–4.25)). There was also a statistical difference in lung consolidation scores according to the severity of pleurisy (*p* value: 0.0023), with a difference between the means of the pleurisy score 0 (2.79 ± 1.88) and scores 2, 3, and 4 (7.29 ± 4.12, and 8.17 ± 4.76, and 7.64 ± 5.37, respectively).

The results demonstrated the presence of DNA in *M. hyopneumoniae* in all collected lung samples (*n* = 70). The mean Ct obtained was 26.69, without statistical difference when comparing the different severities of pleurisy (*p* value: 0.25).

### 3.2. Characterization of the Allelic Profiles of M. hyopneumoniae

**Figure 2:**
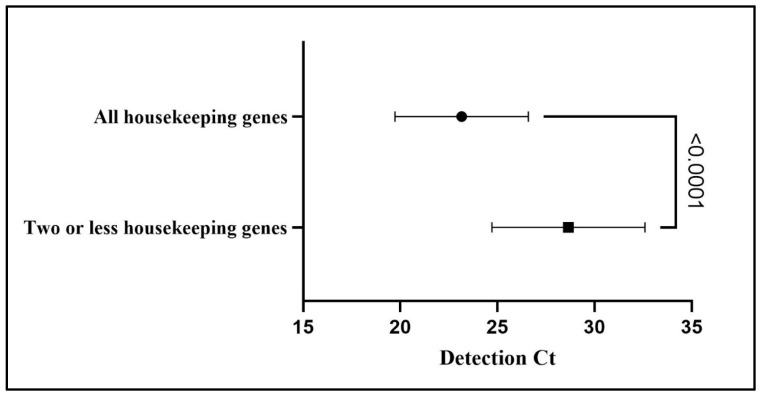
Means and standard deviations of *M. hyopneumoniae* detection Cts in qPCR targeting the p102 gene fragment. Values of samples eligible or not for application of the MLST technique are compared (*p* < 0.05).

The three housekeeping genes (*adk*, *rpoB*, and *tpiA*) were detected in 25 of the 70 samples positive for *M. hyopneunoniae* in the qPCR (35.71%). The mean Ct (23.16 ± 3.43) for detection of the p102 gene of *M. hyopneumoniae* was statistically lower (*p* value: <0.0001) in the samples where it was possible to amplify the three genes required to apply the MLST technique, compared with the samples where only one or two of the maintenance genes were amplified (28.65 ± 3.93) and the MLST technique could not be applied. Figure 2 shows the means and standard deviations of the Cts of the lung samples targeting the p102 gene fragment eligible for the MLST technique.

All sequences corresponded to the allelic types previously deposited in the public database. Three allelic types were identified for the *adk* gene: 6, 13, and 23. For the *rpoB* gene, alleles 11, 18, and 33 were identified. For the *tpiA* gene, the alleles observed were 19, 26, 41, 60, 62, 79, and 88.

**Figure 3:**
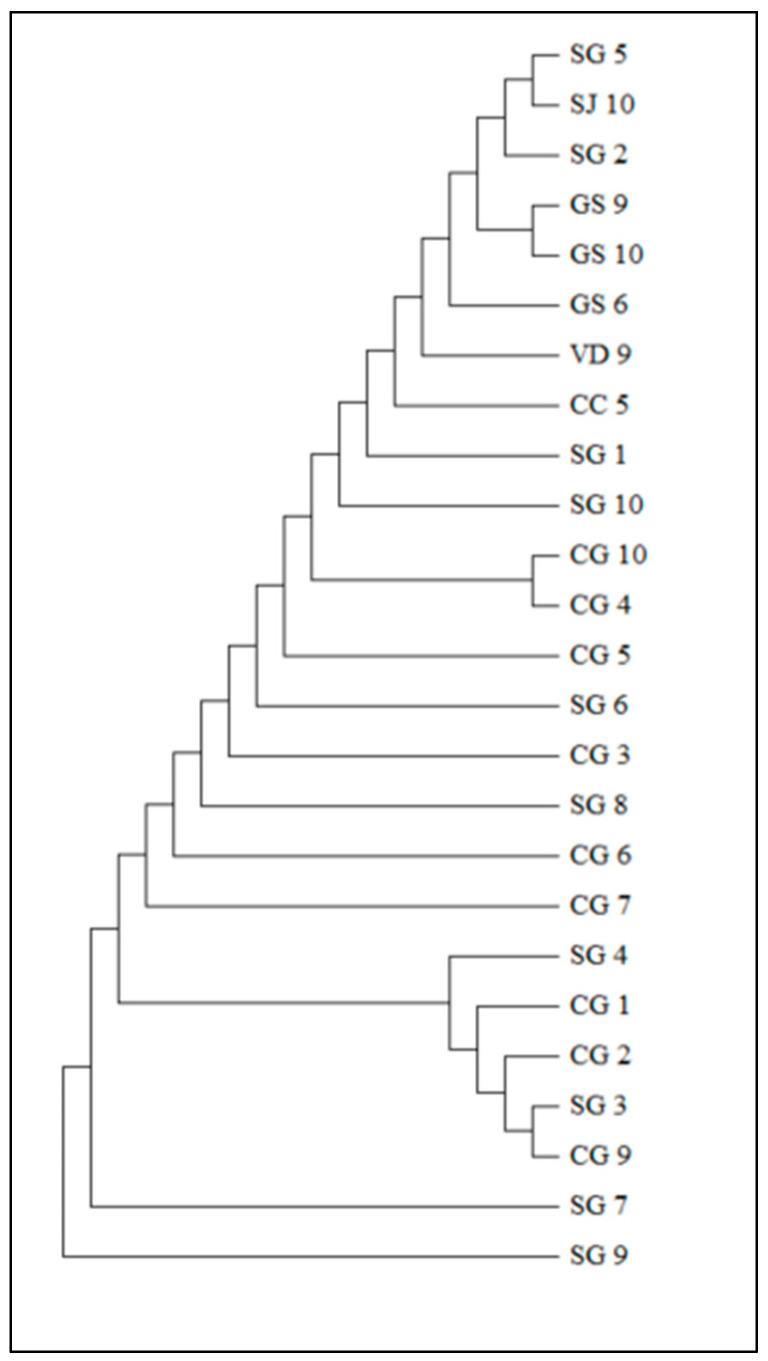
Dendrogram created using the neighbor joining method, based on the sequence of the concatenated genes following the order *adk*, *rpoB*, and *tpiA*. The distances were computed using the *p*-distance method and are expressed as the number of base differences per site.

When these alleles were combined (with respect to the order of description of the target genes: *adk*, *rpoB* and *tpiA*), 10 different allelic profiles were observed as described in Table 1. The allelic profiles found did not correspond to any sequence type already reported in the public database. Figure 3 shows the phylogenetic tree based on the sequence of the concatenated genes. The tree can be divided into 16 clades. Shorter branches were observed in the relationship between CG 9 and SG 7 as well as CG 1 and SG 4. These samples originated from the same producing state (Mato Grosso). However, sequences SG 5 and SJ 10 formed an extremely close pair, suggesting a high degree of similarity, even though their geographical origins are far apart.

Regarding the geographical distribution, the most prevalent allelic profile (23-18-26, found in 56% of the samples (14/25)) was exclusively observed in lung lesion samples from the central west region. Additionally, allelic profiles 23-18-41, 13-18-26, 23-18-60, and 23-11-26 were also found in this region. None of these allelic profiles were found in the southern region of Brazil, where the following profiles were observed: 13-18-79, 13-18-88, 23-33-62, and 6-11-41. Moreover, a distinct profile from the other regions was identified in the southeast region (23-11-19). Figure 1 shows the allelic profiles per state.

### 3.3. qPCR for the Detection of Bacterial Co-Infection

In the detection of co-infection agents associated with pleurisy lesions, of the 25 samples submitted to qPCR, 8 were positive for *Pasteurella multocida* type A (PM) (32%), 10 were positive for *Actinobacillus pleuropneumoniae* (APP) (40%), and 3 were positive for both agents (12%). The qPCR Cts are summarized in Table 2.

### 3.4. Severity of Gross and Histopathological Lesions

Regarding the pleurisy scores assessed at slaughter, based on the genetically characterized samples (*n* = 25), 4 lungs (16%) were classified with pleurisy scores 0 and 1, 6 lungs (24%) with score 2, 3 lungs (12%) with score 3 and 8 lungs (32%) with score 4. Among these, score 0 showed 4 different allelic profiles, scores 1 and 3 had only 1 profile each, score 2 showed 4 different profiles, and score 4 presented 5 different allelic profiles. Table S4 summarizes the macroscopic findings according to allelic profile found in this study, including the mean number of pulmonary consolidation points seen in the macroscopic evaluation and the pleurisy scores.

In the microscopic analysis, all lungs showed histological lesions characteristic of *M. hyopneumoniae* infection (different scores of BALT hyperplasia). Secondary bacterial lesions were frequent, with histological findings of bronchopneumonia or pleuropneumonia observed in 14 out of 25 lungs evaluated (56%). Additionally, histological findings suggestive of sVIA co-infection were observed in 52% (13/25) of the samples, mainly proliferative or obliterative bronchiolitis, which were negative on IHC (*n* = 13). Table 3 describes the severity of the microscopic lesions according to each allelic profile observed in this study.

## 4. Discussion

The allelic profile analysis identified notable genetic diversity among the samples, with different allelic profiles geographically distributed, suggesting possible regional variations in the epidemiology of the infection. The housekeeping genes *(adk*, *rpoB*, and *tpiA*) were detected in 35.71% of the samples (25/70). The success rate for using the technique is challenging. Lung samples, especially those from animals with chronic lesions, often contain a low bacterial load. MLST is a technique which requires amplification of multiple loci and subsequent sequencing. This can be difficult to perform with clinical samples, where the quantity and quality of the pathogen’s DNA is limited.

In a study carried out by Zhang et al. (2021) [27], in 47 out of 199 samples positive for *M. hyopneumoniae* in the nested PCR, it was possible to use the MLST technique. The MLST technique usually requires the amplification of seven target genes, but for *M. hyopneumoniae*, the use of three target genes is sufficient to understand the relationship between the strains. Mayor et al. (2008) [13] demonstrated in her study that three genes had the same resolution when seven genes are used in MLST analysis for *M. hyopneumoniae*.

As the samples came from clinical specimens and not isolates, the presence of inhibitors and a greater amount of genetic material could hinder the performance of the technique [27]. However, considering the complexity and time involved in cultivating and isolating this agent, MLST is a useful tool with high discriminatory power and rapid execution for characterizing *M. hyopneumoniae* and understanding infection dynamics [10,13].

Different strains of *M. hyopneumoniae* can lead to variable clinical signs and severity of lesions. In addition, a single batch of pigs can be infected by multiple strains with different genetic profiles [28,29]. This highlights the importance of studies which aim to correlate the genetic characterization of strains with the severity of lesions for understanding both the epidemiological scenario and the potential response to vaccine antigens. In the present study, regardless of the observed allelic profile, the sampled animals exhibited histopathological lesions consistent with *M. hyopneumoniae* infection, with varying scores of pleurisy severity. However, we did not perform any correlations since the number of samples per allelic profile found was too limited.

Our study identified 10 new combinations of alleles not yet described in the PubMLST database, but it was not possible to categorize the STs. This could have been because isolates with these genetic characteristics have not yet been described. Considering that only one other study has been carried out in Brazil using this technique, we can suggest that the allelic profiles observed in this study are characteristic of Brazil. Research in other countries, such as China, has also shown great genetic variation in *M. hyopneumoniae*, where 16 new sequence types were found in 47 sequenced samples [27]. For a new ST to be incorporated into the database, data from an isolate or a complete genome are required (https://pubmlst.org/submit-data, accessed on 16 July 2024). The possible presence of multiple strains in the clinical sample can generate artifacts and difficult data analysis. Therefore, a careful evaluation of the electropherograms is essential to guarantee the quality of the results found. This underscores the need to increase efforts to characterize the new strains currently circulating. Based on this, we can hypothesize that the samples belong to sequence types characteristic of Brazil, especially given that only two Brazilian isolates are currently documented in the database.

Additionally, the present study investigated the relationship between lung consolidation, the presence of *M. hyopneumoniae* DNA, and allelic profiles in lung samples from pigs with macroscopic lesions consistent with *M. hyopneumoniae* infection in Brazilian herds. Our findings revealed a significant correlation between the severity of pleurisy lesions and the lung consolidation scores, indicating that *M. hyopneumoniae* infection may play a critical role in the pathogenesis of the observed lung lesions. *M. hyopneumoniae* presented high prevalences of 92.45% and 97.7%, as reported in previous studies conducted in Brazil [4,8]. Marois et al. (2008) [30] demonstrated that cross-contamination can occur in the scalding tanks at slaughterhouses, but in our study, the macro- and microscopic lesions supported *M. hyopneumoniae* infection. This finding reinforces the relevance of *M. hyopneumoniae* as a primary pathogen in the complex of respiratory system diseases in pigs [31].

Co-infections between *M. hyopneumoniae* and PM (32%) and *M. hyopneumoniae* and APP (40%) emphasize the complexity of swine respiratory infections, where several pathogens may be involved. Arruda et al. (2024) found that 10.1% of cases exhibited co-identification involving *M. hyopneumoniae*, PM, and APP [8]. This percentage is similar to the 12% found in this study. The presence of co-infections can aggravate the severity of the disease, influence the response to treatment, and complicate control efforts [32]. In our study, no correlations were observed between the severity of the lesions and the presence of co-infections detected by qPCR. The clinical course in the pigs at farms is unknown since the samples were obtained at a slaughterhouse.

Furthermore, approximately half of the samples (13/25; 52%) exhibited chronic microscopic lesions suggestive of sIVA infection, even though they tested negative under IHC examination. However, chronic histological lesions caused by sIVA are typical, and negative IHC results during this stage of infection are common, as reported by Menegatt et al. (2023) [33]. Additionally, it was noted that these samples had statistically lower Ct detection for *M. hyopneumoniae* (*p* value: <0.005). This can suggest a possible exacerbation of *M. hyopneumoniae* infection after lesions caused by sIVA, since previous sIVA infections can facilitate colonization of the respiratory tract by *M. hyopneumoniae* [34].

The sample size was low and could therefore be considered a limitation in this study. The MLST technique requires sufficient lung samples, and more animals per batch or parameter are needed to correlate the prevalence of a determined *M. hyopneumoniae* allelic profile. However, we can suggest that there is a great genetic diversity of *M. hyopneumoniae* strains circulating in Brazilian herds.

## 5. Conclusions

We found a high prevalence of *M. hyopneumoniae* when using qPCR in lung samples with pneumonia lesions and different pleurisy scores. In addition, the most severe pleurisy lesions also had a higher lung consolidation score. BALT hyperplasia lesions associated with *M. hyopneumoniae* infection were the most prevalent microscopic findings. This study suggests a maximum Ct (28.65 ± 3.93) for the detection of *M. hyopneumoniae* in qPCR using the *p102* gene to target triage samples for the MLST technique. The allelic profile analysis identified notable genetic diversity among the samples, with different allelic profiles distributed in different regions of Brazil, suggesting possible regional variations in the epidemiology of the infection. These results contribute to a better understanding of the dynamics of *M. hyopneumoniae* infection in pigs and highlight the importance of considering both pathogen genetics and the macroscopic and microscopic characteristics of lesions when assessing disease severity. For a better epidemiological understanding and future comparisons of the genetic profile of the strains, more isolates of the agent should be characterized.

## Figures and Tables

**Table 1:** Allelic profiles found in this study (following the order *adk*, *rpoB*, and *tpiA*) and their frequencies (percentage and absolute number).

Allelic Profiles	Samples
23-18-26	56% (14/25)
23-18-41	8% (2/25)
13-18-79	8% (2/25)
13-18-26	4% (1/25)
23-18-60	4% (1/25)
13-18-88	4% (1/25)
23-11-19	4% (1/25)
23-33-62	4% (1/25)
6-11-41	4% (1/25)
23-11-26	4% (1/25)

**Table 2:** Pig batch origin (city and state), allelic profile, pleurisy score, detection of Cts of *Mycoplasma hyopneumoniae* (Mhyo), *Actinobacillus pleuropneumoniae* (APP), and *Pasteurella multocida* type A (PM), and presence of microscopic lesions like sIVA infection * for each sampled lung.

Sample	Herd Origin	MLST Allele Profile			Pleurisy Score	Mhyo Ct	APP Ct	PM Ct	Lesions Like sIVA Infection
		*adk*	*rpoB*	*tpiA*					
CG 1	Campo Grande (MS)	23	18	26	0	23.79	30.73	ND	No
CG 3	Campo Grande (MS)	23	18	26	1	18.59	38.00	ND	Yes
CG 4	Campo Grande (MS)	23	18	26	1	26.33	29.22	ND	Yes
SG 3	São Gabriel do Oeste (MS)	23	18	26	1	23.39	ND	ND	No
SG 4	São Gabriel do Oeste (MS)	23	18	26	1	30.27	ND	ND	No
CG 5	Campo Grande (MS)	23	18	26	2	28.54	31.71	ND	No
CG 6	Campo Grande (MS)	23	18	26	2	18.82	ND	ND	Yes
SG 6	São Gabriel do Oeste (MS)	23	18	26	2	22.76	ND	ND	Yes
CG 7	Campo Grande (MS)	23	18	26	3	21.78	27.96	ND	No
SG 7	São Gabriel do Oeste (MS)	23	18	26	3	20.43	ND	ND	No
SG 8	São Gabriel do Oeste (MS)	23	18	26	3	23.45	35.20	ND	Yes
CG 9	Campo Grande (MS)	23	18	26	4	17.24	29.34	23.69	Yes
SG 9	São Gabriel do Oeste (MS)	23	18	26	4	21.35	ND	ND	Yes
SG 10	São Gabriel do Oeste (MS)	23	18	26	4	22.27	ND	ND	Yes
SG 2	São Gabriel do Oeste (MS)	23	18	41	0	18.24	ND	ND	Yes
SG 5	São Gabriel do Oeste (MS)	23	18	41	2	25.65	ND	28.60	Yes
GS 9	Guarujá do Sul (SC)	13	18	79	4	21.52	ND	23.48	Yes
GS 10	Guarujá do Sul (SC)	13	18	79	4	24.68	ND	27.12	No
CG 2	Campo Grande (MS)	13	18	26	0	24.94	29.00	ND	No
SG 1	São Gabriel do Oeste (MS)	23	18	60	0	27.04	ND	ND	Yes
GS 6	Guarujá do Sul (SC)	13	18	88	2	25.00	ND	27.82	Yes
CC 5	Cerqueira César (SP)	23	11	19	2	29.06	30.84	29.24	No
VD 9	Videira (SC)	23	33	62	4	22.29	ND	36.92	No
SJ 10	São José do Cedro (SC)	6	11	41	4	20.74	ND	ND	No
CG 10	Campo Grande (MS)	23	11	26	4	20.83	20.72	17.46	No

* Bronchiolitis (necrotic, proliferative, or obliterative).

**Table 3:** Histopathological findings in the 25 lung samples and for each allelic profile. The results are presented as percentages and absolute numbers.

Histological Lesion	Total	23-18-26	23-18-41	13-18-79	13-18-26	23-18-60	13-18-88	23-11-19	23-33-62	6-11-41	23-11-26
BALT *											
0	-	-	-	-	-	-	-	-	-	-	-
+	28 (7/25)	28.57 (4/14)	-	100 (2/2)	-	-	-	100 (1/1)	-	-	-
++	56 (14/25)	57.14 (8/14)	50 (2/2)	-	100 (1/1)	100 (1/1)	100 (1/1)	-	-	100 (1/1)	-
+++	16 (4/25)	14.28 (2/14)	-	-	-	-	-	-	100 (1/1)	-	100 (1/1)
Suppurative bronchopneumonia	24 (6/25)	7.14 (1/14)	50 (1/2)	50 (1/2)	-	-	100 (1/1)	-	100 (1/1)	-	100 (1/1)
Necrotizing bronchopneumonia	24 (6/25)	28.57 (4/14)	50 (1/2)	-	100 (1/1)	-	-	-	-	-	-
Pleuropneumonia	8 (2/25)	7.14 (1/14)	-	-	-	-	-	-	-	-	100 (1/1)
Chronic pleuritis	80 (20/25)	78.57 (11/14)	50 (1/2)	100 (2/2)	-	100 (1/1)	100 (1/1)	100 (1/1)	100 (1/1)	100 (1/1)	100 (1/1)
Pneumocyte hyperplasia	84 (21/25)	85.71 (12/14)	100 (2/2)	50 (1/2)	100 (1/1)	100 (1/1)	100 (1/1)	-	100 (1/1)	100 (1/1)	100 (1/1)
Alveolar edema	28 (7/25)	21.43 (3/14)	50 (2/2)	50 (1/2)	-	-	100 (1/1)	-	-	-	-
Lesions like sIVA infection **	52 (13/25)	57.14 (8/14)	100 (2/2)	50 (1/2)	-	100 (1/1)	100 (1/1)	-	-	-	-

* BALT = bronchial-associated lymphoid tissue hyperplasia. BALT hyperplasia was classified as absent (0), mild (+), moderate (++), or extensive (+++). ** Bronchiolitis (necrotic, proliferative, or obliterative).

## Data Availability

The original contributions presented in the study are included in the article/Supplementary Materials, further inquiries can be directed to the corresponding author.

## Supplementary Materials

The following supporting information can be downloaded at https://www.mdpi.com/article/10.3390/microorganisms12101988/s1. Table S1: Sequences of the primers and hydrolysis probes used for each target region of the multiplex qPCR: *omIA* gene (APP), *p102* gene (*M. hyopneumoniae*), and *kmt1* gene (PM), and the respective amplifier sizes (bp). Table S2: Sequences of the primers for *M. hyopneumoniae* MLST (amplification and sequencing). Table S3: Data for each lung evaluated. Table S4: Mean number of pulmonary consolidation points, with total for each lung evaluated and per lung lobe assessed in the macroscopic evaluation and the number of lungs per pleurisy score according to the allelic profile.
